# Harm Reduction as a Form of ‘Wrap‐Around’ Care: The Nursing Role

**DOI:** 10.1111/inm.13436

**Published:** 2024-09-20

**Authors:** Marissa D. Abram, Adrian Jugdoyal, Paulo Seabra, Dana Murphy‐Parker, Adam Searby

**Affiliations:** ^1^ Duke University School of Nursing Durham North Carolina USA; ^2^ Brunel University London UK; ^3^ Nursing School of Lisbon Lisbon Portugal; ^4^ University of Colorado Anschultz Medical Center Aurora Colorado USA; ^5^ Monash University School of Nursing and Midwifery Melbourne Victoria Australia

**Keywords:** addiction treatment, harm minimization, harm reduction, nurse's role, nursing

## Abstract

In addiction treatment, harm reduction is a philosophy that aims to reduce the harms from ongoing alcohol and other drug use. Although abstinence may be the ‘gold standard’ in reducing harm from ongoing alcohol and other drug use, harm reduction recognises that abstinence may not be achievable for certain individuals. Accordingly, harm reduction is used to enable medical or mental health treatment for individuals who continue to use alcohol and other drugs, providing a form of care which meets individuals where they present to healthcare facilities. Harm reduction accepts ongoing alcohol and other drug use, while providing a traditionally marginalised cohort of individuals access to healthcare services. In this perspective paper, we argue that the role of nurses in promoting and utilising harm reduction as part of their regular practice is essential to both reducing harm from alcohol and other drug use, engaging individuals who use alcohol and other drugs in healthcare services, and providing a means to accept individuals as they are to build trust and rapport for engagement in addiction treatment when they are ready, and at their own pace. Nurses, by virtue of their role and number in the healthcare landscape (approximately 28 million globally), are ideally placed to implement harm reduction in their practice to achieve better outcomes for individuals who use alcohol and other drugs.

## 1 Introduction

Harm reduction, historically is defined as an approach that ‘… attempts to reduce the adverse consequences of drug use among persons who continue to use drugs’, (Single 1995, 287). Harm reduction has become an adjunct evidence‐based intervention in many global regions providing addiction treatment, particularly to reduce the harms inherent in the continual use of alcohol and other drugs (Marlatt and Witkiewitz 2002). It can be also be utilised for individuals who are not eligible or engaged in addiction treatment services. For example, harm reduction has been recognised as a viable option for cohorts who use drugs and may not seek treatment, such as pill testing with recreational users of 3,4‐methylenedioxy methamphetamine (MDMA) and associated strategies to reduce harm for casual users of these substances (Southey et al. 2020).

Drug and alcohol use occurs frequently and along a continuum, from abstinence, through experimental and recreational, to dependent and harmful use. Approximately 296 million individuals used drugs in 2021 and 39.9 million of those individuals met criteria for a drug use disorder globally (United Nations, Office on Drugs and Crime 2023). For the estimated 2.3 billion individuals using alcohol in the world, about 300 million people experienced an alcohol use disorder (World Health Organization 2018, 2023). The aforementioned recreational MDMA user sits at the ‘non‐dependent’ end of the continuum, whereas those who use alcohol and drugs in a daily, dependent fashion tend to sit at the opposite end of the continuum, and are most likely to seek care from addiction treatment services (Andrews et al. 2012; Askew 2016). However, less than 20% of people who need treatment access due to systemic barriers. Furthermore, treatment does not readily translate to abstinence. Several studies indicate rates of abstinence 12 months after treatment in the range of 32% (Williams and Chang 2000) to 71% (Greenfield et al. 2004), indicating a need for harm reduction approaches for individuals who do not achieve success in addiction treatment and continue to use alcohol and other drugs and for those with experimental and recreational use. The majority of individuals with alcohol and drug use are not in addiction treatment and if harm reduction is not implemented across all healthcare settings, it is a missed opportunity to reduce risk and minimise mental and physical health consequences for these individuals.

## 2 Aim

The aim of this paper is to outline this position with reference to the evidence for harm reduction, and to argue that harm reduction needs to be implemented into mainstream healthcare settings to reduce the personal and societal burden of problematic alcohol and other drug use.

## 3 Background

Harm reduction provides a range of health and social services that focus on mitigating the negative consequences of alcohol and/or other drug use by providing compassionate care and promoting the autonomy of the individual (Substance Abuse and Mental Health Services Administration [SAMHSA] 2023). Through services such as supervised injection centres, education on safer use, overdose prevention and drug checking, harm reduction saves lives by preventing drug related deaths and disability. Furthermore, other services such as syringe exchange programs can reduce the risk of acute bacterial or fungal infection, and other chronic diseases such as human immunodeficiency virus (HIV), viral hepatitis and bacterial or fungal infections (Curado et al. 2022; Marlatt 1996).

Although no intervention can make alcohol or substance use completely safe, harm reduction endeavours to reduce preventable harms to individuals who use alcohol and other drugs. Often, this is an approach used to those who are in Prochaska and DiClemente's (1983) stages of pre‐contemplation or early contemplation, and is a useful tool to reduce risks and harms while working with individuals to engage them in treatment for alcohol and other drug use, mental health, physical health or psychosocial issues that may be detrimental to their wellbeing (Bertrand et al. 2015). An example of a harm reduction intervention that allows treatment engagement is medically supervised injecting or drug consumption rooms; examples from the two rooms operating in Australia indicate that in the Sydney injecting room, there was an uptake of referrals for substance use, health care and psychosocial referrals of 35% (Kimber et al. 2008), and the facility in Melbourne reporting approximately 80% of individuals using the facility accessing at least one form of health, wellbeing or social support service (Centre for Evaluation and Research Evidence 2023). The wider community also benefited from supervised consumption rooms, as there were less accidental deaths and overdoses as well as a reduction of injections in public spaces (Van den Boom et al. 2021).

The preceding figures indicate the true value of harm reduction in providing care to individuals where they present, regardless of their status of alcohol or other drug use. For example, experimental or recreational users having their pills checked at a music festival may not necessarily seek treatment. Accordingly, Santamarina et al. (2024) argue that deaths from recreational substance use could likely be prevented ‘… through the implementation of a range of harm reduction strategies, including mobile medical care, drug checking services, and increased consumer education and awareness’, (p. 1).

Harm reduction approaches have been shown in research to be acceptable to individuals who use alcohol and other drugs. For example, Reed et al. (2021) explored the acceptability of fentanyl test strips in Philadelphia, United States, interviewing 15 individuals who used heroin and crack cocaine, finding a high level of knowledge of the threat and severity of fentanyl adulteration, an understanding of the perceived benefits of drug checking, and an acceptability of taking the step to check drugs prior to administration. Historically, these results mirror those found for earlier harm reduction initiatives, such as needle exchanges, which have been both shown to prevent blood borne viruses and have almost universal uptake among individuals who inject substances (Belani and Muennig 2008; Neaigus et al. 2008; Ritter and Cameron 2006).

In addition to excess morbidity and premature mortality, alcohol and/or drug use can have a significant economic toll on society. It is estimated that the opioid epidemic in the United States cost 1.5 trillion US dollars in 2020, a number that is expected to continue trending up as drug overdose rates continue to increase (Heinrich 2022). Cost analysis studies and estimated cost models demonstrate that harm reduction interventions such as supervised injection sites are cost effective strategies that reduce the economic burden and improve health outcomes of individuals with alcohol and/or drug use (Chambers et al. 2022; Hood et al. 2019; Khair et al. 2022).

## 4 Design

In this perspective paper, we argue that the role of nurses in promoting and utilising harm reduction as part of their regular practice is essential to both reducing harm from alcohol and other drug use, engaging individuals who use alcohol and other drugs in healthcare services, and providing a means to accept individuals as they are to build trust and rapport for engagement in addiction treatment when they are ready, and at their own pace. Nurses, by virtue of their numbers and presence in healthcare systems, are ideally placed to implement harm reduction approaches in three domains: (1) accepting that there are individuals who enter healthcare systems may be unable, not ready or not planning to achieve abstinence, (2) meeting individuals who use alcohol and/or other drugs where they present, (3) providing ‘wrap‐around’ integrated healthcare for individuals who use alcohol and/or other drugs.

## 5 Method

In this paper, we critically explore three domains that we perceive are necessary for nurses to adopt harm reduction for individuals who use alcohol and/or other drugs, regardless of healthcare setting. Following this exploration, we provide three recommendations we believe are necessary for nurses to adopt harm reduction in their everyday practice.

### 5.1 Accepting That There Are Individuals who Enter Healthcare Systems May Be Unable, Not Ready or Not Planning to Achieve Abstinence

Harm reduction has historically recognised that there are individuals who are either unable to attain abstinence for their alcohol and/or other drug use, therefore, harm reduction aims to accept individual substance use while providing strategies to reduce harm from that use (Marlatt 1996). Such examples include medically supervised injecting or drug consumption rooms, ‘allowing’ the use of substances in order to provide immediate response in the case of overdose, preventing cognitive impairment through hypoxia or respiratory arrest that is common in overdose, particularly with opioids (Roxburgh et al. 2017).

Mainstream healthcare settings often see individuals who are unable, not ready, or not planning to work towards abstinence; they may have been admitted for an injury, illness or mental health concern that is their primary focus. Nurses often need to interact with individuals who have entered healthcare systems for reasons other than their alcohol and/or other drug use, and these encounters should be considered an opportunity to provide harm reduction education. Musalek (2013) argues that harm reduction needs to form part of a dynamic approach to addiction treatment, and that focusing solely on abstinence‐based approaches to addiction treatment are not effective in clinical practice.

As a treatment paradigm, harm reduction has been implemented in hospitals globally. For example, Hyshka et al. (2019) explored patient perspectives on an addiction medicine service in a Western Canadian hospital, which used a harm reduction approach designed to address high rates of those leaving against medical advice and premature discharge, which ultimately led to unplanned readmissions. The service employed harm reduction approaches including distribution of injecting equipment, provision of naloxone kits and safer drug use education, as well as connections to social and health services where required. Patient feedback reported this approach as non‐judgemental and supporting their autonomy, with positive regard for the service described among substance using peers.

Given the size of the workforce, nurses are ideally placed to provide harm reduction advice to individuals who use alcohol and other drugs, particularly when they present to healthcare services for issues that are not related to AOD use. A condition of nursing care should not be abstinence from alcohol and/or drugs, and there is an argument that nurses have an obligation to provide healthcare to individuals regardless of whether they continue to use alcohol and/or other drugs (Abram, Seabra, and Searby 2023). There is no wrong entry point in the healthcare system to provide harm reduction interventions and all individuals deserve safe, high‐quality care regardless of their substance use status.

### 5.2 Meeting Individuals who Use Alcohol and/or Other Drugs Where They Present

Frequently, individuals who use alcohol and other drugs do not present to traditional healthcare services for several reasons; stigma towards their alcohol or other drug use may hinder help seeking behaviour, they may have limited access to care, traditional clinic models may not take into account comorbid mental or physical ill health, or they may not have the financial means to pay provider bills (Dunlop et al. 2020; Room 2005). A survey study of 246 individuals seeking emergency department care who use substances in Canada highlights this point, with participants reporting that they were frequently considered drug seeking and subject to judgemental and untimely care (Rajab et al. 2023).

The notion of meeting individuals who use alcohol and other drugs where they present is not new and has been used to great effect in medically supervised drug consumption rooms globally. In addiction treatment, this approach has been used to allow individuals to enter treatment when they are ready; for example, a review of the Medically Supervised Injecting Room in Sydney, Australia, found that 64% of frequent attenders (defined as those with over 98 total visits) accepted a referral to drug treatment during the period 2001–2010. As the report indicates, ‘Overall, the more frequently the client attended [the injecting room], the more likely they were to accept a referral’, (KPMG 2010, 25). Similar results are noted in Portugal, where a mobile supervised injecting facility not only meets individuals where they present, but affords an opportunity to provide treatment for the approximately 70% of hepatitis C injecting drug users that use the facility (Curado et al. 2022).

Meeting individuals where they present allows for non‐judgemental, humanistic care where the focus of the episode is not only alcohol and/or other drug use but a holistic view of individual circumstances. Qualitative research studies have demonstrated that positive staff attitudes towards individuals who use alcohol and other drugs who present to healthcare services lead to greater engagement and satisfaction with services (Branson et al. 2022), and provide a sense of dignity to those who continue to use drugs (Kappel et al. 2016).

### 5.3 Providing ‘Wrap Around’ Integrated Healthcare for Individuals who Use Alcohol and/or Other Drugs

Wrap‐around services are defined as ‘… health care and social services that, when applied with substance use treatment, are intended to improve client access and retention within substance use treatment as well as to address the comprehensive and treatment‐specific needs of clients’, (Pringle et al. 2002, 110). Although this definition speaks of substance use treatment, we argue that a wrap‐around model of care is valuable in situations where individuals continue to use alcohol and/or other drugs to address the mental health, physical health and psychosocial comorbidities associated with alcohol and other drug use.

The concept of wrap‐around care is particularly relevant when providing services to individuals who use alcohol and/or other drugs; for example, the Medically Supervised Injecting Room in Melbourne, Australia provided 19743 linkages to support services, with 80% of these to health promotion services and injecting‐related injuries, during a two year period to individuals who continued to inject drugs (Victorian Government 2023). Prior to this, needle exchange programs have incorporated wrap‐around care into their remit. For example, Owens et al. (2020) report on a pilot program reported on the implementation of a trauma sensitive reproductive health program for women attending the needle exchange for injecting equipment, with clients of the service (*n* = 15) reporting the accessibility and walk in nature of the service as a significant benefit and reason to use it, among others. It also provides the opportunity to monitor high risk injecting drug users who may inject in the groin or using cocaine intravenously. This is also provide instructions on wound care and signs and risks of overdose (Shorte et al. 2022).

Wrap‐around care can be bidirectional and has been shown to be successful in other areas; for example, Goldbach and Kipke's (2022) qualitative study of Black and Latino HIV‐positive men who have sex with other men (*n* = 15) in the United States found that greater satisfaction with care was described where services not only focussed on HIV, but provided services such as dental and mental health. Treatment efficacy has also been shown with an intervention for homeless veterans with co‐occurring disorders, where 218 of the overall 333 enrolled received a wrap‐around care intervention and at 12‐month follow up were experiencing less mental health symptoms and were less likely to consume alcohol to intoxication (Smelson et al. 2013).

## 6 Recommendations

### 6.1 Harm Reduction Should Be Integrated Into Nursing Education

Harm reduction is a philosophy, which is designed not to make alcohol and/or drug use safe, but to minimise the harms that occur (Single 1995). For harm reduction to reach this aim, nurses need to be educated not only in its philosophies, but how to apply it across the spectrum of alcohol and other drug use and all healthcare settings. Rather than demand abstinence as a cornerstone to treatment for mental or physical ill health, harm reduction approaches should be taught and tailored to providing healthcare to the most marginalised populations. If an individual is unable, not ready or not planning to make changes to their alcohol and/or other drug use it should not preclude them from receiving healthcare, nor should it viewed as a moral failing and a reason to blame them for their ‘poor choices’, (Searby, Burr, and Abram 2024; Tsai et al. 2019).

An extension is that healthcare should be provided to individuals who use substances at the experimental or recreational end of the spectrum. Rather than having a ‘just say no’ philosophy, nurses should be provided education on how to reduce harm for this group. This should include providing information on initiatives such as drug checking, providing information and education on the dangers of consuming substances with alcohol, and polysubstance use. Research has shown that harm reduction education is sorely lacking for nurses. For example, Watts et al. (2023) found that only 17% of surveyed nurses had been taught about harm reduction in their preregistration nursing program. Although this study comes with the caveat of a small sample size (*n* = 30), similar work indicates that education on alcohol and other drugs is seldom provided (Searby and Burr 2020).

In the UK, individuals who have lived experienced have been involved in the development, teaching and assessment of nursing in undergraduate education. This has been seen as beneficial in destigmatising language and providing knowledge of medical conditions and should be done when introducing harm reduction principles to nurses. This results in the acquisition of new knowledge and skills, gaining confidence in awareness, building better relationships and improving the human interaction approach to care (O'Connor et al. 2021).

### 6.2 Harm Reduction Needs to Be a Component of the Comprehensive Nursing Assessment

When assessing individuals who use alcohol and/or other drugs, nurses need to be able to provide strategies to engage individuals in treatment and reduce the harms of ongoing alcohol and/or drug use. These strategies may include options such as the provision of take‐home naloxone, instructions on how to access injecting facilities, advice on where to find drug checking services or providing education on driving under the influence of drugs and/or alcohol. The assessment provides an opportune time to screen for risk, provide information on strategies to reduce harm, and support individuals by offering information on how to access these services. A shift in the nursing assessment and treatment paradigm is urgently required. At present, there are a subset of nurses who describe individuals, particularly those who are opioid dependent, as ‘drug seeking’ and not worthy of interventions that non‐opioid dependent individuals receive, primarily pain relief (Febres‐Cordero, Shasanmi‐Ellis, and Sherman 2023). Harm reduction is health promotion and disease prevention for individuals who use substances, which is a core element of the nursing role.

### 6.3 Stigma Towards People who Use Alcohol and/or Drugs Should Be Addressed Among Nurses

Stigma towards individuals who use alcohol and/or other drugs is prevalent among healthcare professionals including nurses and negatively impacts the quality of care they receive (Cazalis et al. 2023; Van Boekel et al. 2013). The harmful discrimination and mistreatment experienced during healthcare interactions leads to consequences should as delays in seeking and receiving treatment, prevents individuals from disclosing health information such as alcohol and/or other drug use and minimalising symptoms such as pain to be more credible to the provider (Biancarelli et al. 2019).

Stigma can and must be address in mainstream healthcare setting among nurses. Specifically factors that contribute to alcohol and/or other drug use stigma that are modifiable are: the view of addiction as a moral failing, negative beliefs about individuals who use alcohol and/or other drugs, lack of training, time, and role support for nurses (Cazalis et al. 2023). Additionally, nurses must be educated about the value of harm reduction as an evidence‐based health promotion and disease prevention approach that decreases mortality and morbidity, increases treatment engagement, and creates a pathway towards wellness and recovery (Addison 2023; Substance Abuse and Mental Health Services Administration 2023).

## 7 Conclusions

Nurses are critical in implementing harm reduction services as a wrap‐around model of care into mainstream healthcare. However, to make this paradigm shift, they need to be educated and trained at the undergraduate level to embed harm reduction into clinical practice. Furthermore, for harm reduction strategies to be implemented successfully, the stigma towards people who use alcohol and/or drugs must be addressed among nurses. Only then will nurses across healthcare settings, be prepared to reduce risk, and provide safe, high‐quality care for the person entering healthcare who may not be able or ready to stop using substances.

## 8 Relevance for Clinical Practice

Individuals with alcohol and/or other drug use experience harmful healthcare interactions and outcomes due to stigma and negative interactions with the healthcare system. When these individuals present to mainstream health care settings, nurses can provide compassionate care, re‐build trust and re‐engage them in care. Harm reduction interventions ‘meet individuals where they are at’, in an accepting, non‐judgmental, respectful manner while providing treatment for the presenting concern. It is an evidence‐based approach that promotes health, prevents disease, and reduces risk related to alcohol and/or other drug use.

## Author Contributions

Each author certifies that their contribution to this work meets the standards of the International Committee of Medical Journal Editors.

## Conflicts of Interest

The authors declare no conflicts of interest.

## Data Availability

Data sharing not applicable to this article as no datasets were generated or analysed during the current study.
